# High incidence of radiation pneumonitis in lung cancer patients with chronic silicosis treated with radiotherapy

**DOI:** 10.1093/jrr/rrz084

**Published:** 2019-12-10

**Authors:** Tianle Shen, Liming Sheng, Ying Chen, Lei Cheng, Xianghui Du

**Affiliations:** 1 Department of Radiotherapy, Shanghai Chest Hospital, Shanghai Jiao Tong University, Shanghai 20030, China; 2 Department of Radiotherapy, Zhejiang Cancer Hospital, Hangzhou 310022, China

**Keywords:** Lung cancer, chronic silicosis, radiotherapy, dosimetric, radiation pneumonitis

## Abstract

Silica is an independent risk factor for lung cancer in addition to smoking. Chronic silicosis is one of the most common and serious occupational diseases associated with poor prognosis. However, the role of radiotherapy is unclear in patients with chronic silicosis. We conducted a retrospective study to evaluate efficacy and safety in lung cancer patients with chronic silicosis, especially focusing on the incidence of radiation pneumonitis (RP). Lung cancer patients with chronic silicosis who had been treated with radiotherapy from 2005 to 2018 in our hospital were enrolled in this retrospective study. RP was graded according to the National Cancer Institute’s Common Terminology Criteria for Adverse Events (CTCAE), version 3.0. Of the 22 patients, ten (45.5%) developed RP ≥2. Two RP-related deaths (9.1%) occurred within 3 months after radiotherapy. Dosimetric factors V_5_, V_10_, V_15_, V_20_ and mean lung dose (MLD) were significantly higher in patients who had RP >2 (*P* < 0.05). The median overall survival times in patients with RP ≤2 and RP>2 were 11.5 months and 7.1 months, respectively. Radiotherapy is associated with excessive and fatal pulmonary toxicity in lung cancer patients with chronic silicosis.

## INTRODUCTION

Radiotherapy is used for the treatment of almost 75% of lung cancer patients for curative or palliative purpose [1]. In patients with early-stage disease, radiotherapy could offer an outcome comparable to that achieved by surgery but with lower morbidity [2]. In locally advanced tumors, adjuvant radiotherapy increased the absolute 5-year overall survival (OS) by 5%, compared with surgery alone [3]. When patients develop recurrence or metastasis, radiotherapy could also be used as a palliation approach with acceptable toxicities [4]. Radiation-induced pneumonitis (RP) and esophagitis remain dose-limiting complications that affect the widespread use of radiotherapy in lung cancer patients. RP refers to the damage of normal lung tissue caused by radiation, which occurs between 1 month and 1 year after radiotherapy [5]. RP has also been reported to result in poorer quality of life and reduced OS [6]. With the advancement of radiotherapy technology, the incidence of radiation pneumonia has decreased. Intensity-modulated radiation therapy (IMRT) is associated with lower rates of RP, compared with 3D conformal radiation therapy (3D-CRT) [7]. The incidence of severe RP after IMRT is 8–20% [7–10]. So far, dosimetric factors including mean lung dose (MLD), V_5_ and V_20_ have been considered to be the best predictors of RP. However, these lung dose–volume constraints were based on clinical data from patients with lung cancer who were treated with concurrent chemoradiotherapy. These constraints were not strict for patients with abnormal pulmonary function. Abnormal pulmonary function has been found to correlate with increased RP [11, 12]. For lung cancer patients with pulmonary emphysema, asthma and pulmonary fibrosis, more stringent lung dose–volume limits should be proposed when receiving radiotherapy [13–15].

In 1997, crystalline silica was found to be a human group I carcinogen, especially for lung cancer [16]. Due to excessive inhalation of crystalline silica particles, macrophages and neutrophils in lung tissues produce a variety of inflammatory cytokines, chemokines and reactive oxygen species (ROS). Furthermore, ROS cause damage to pulmonary epithelial cells and result in fibrogenesis [17]. Silicosis is one of the most common and serious occupational diseases in the world. There are ~20 000 new silicosis cases per year in China and 4.2% of deaths among Chinese workers is attributed to silica dust exposure [18]. Silicosis is classified into three groups according to exposure duration: acute (<5 years), accelerated (5–10 years) and chronic (>10 years). A recent study has found that chronic silicosis in patients is often accompanied by pulmonary diffusive dysfunction [19]. Therefore, we speculate that lung cancer patients with chronic silicosis are more prone to develop RP after radiotherapy and dose–volume constraints should be stricter for those patients.

We conducted this retrospective study to evaluate the incidence of RP in lung cancer patients with chronic silicosis. We further analyzed the relationship between RP and OS in lung cancer patients with chronic silicosis after radiotherapy.

## MATERIALS AND METHODS

### Patients

The patients with lung cancer and chronic silicosis who had been treated with radiotherapy from 2005 to 2018 in our hospital were enrolled in this retrospective study. Patients without pathological or histological confirmation were excluded. Patients who were treated with radiotherapy with or without chemotherapy were included. Patients who had undergone lung surgery were excluded. Finally, a total of 22 patients were included in the analysis. Pulmonary function tests were performed with spirometry under standardized conditions 1 week before radiotherapy. Forced expiratory volume in one second (FEV1), forced volume capacity (FVC) and diffusion capacity for carbon monoxide (DLCO) were calculated.

Patients’ data, such as gender, age, tumor location, histology, smoking and alcohol use, were extracted from the database. The extent of the tumor was determined by the tumor-node-metastasis (TNM) staging system according to the eighth edition of the Union for International Cancer Control (UICC) classification system. This study was approved by the Zhejiang Cancer hospital Institutional Review Board. Informed consent was obtained from all participants according to the Declaration of Helsinki on Biomedical Research Involving Human Studies.

### Diagnosis of chronic silicosis

**Fig. 1.**
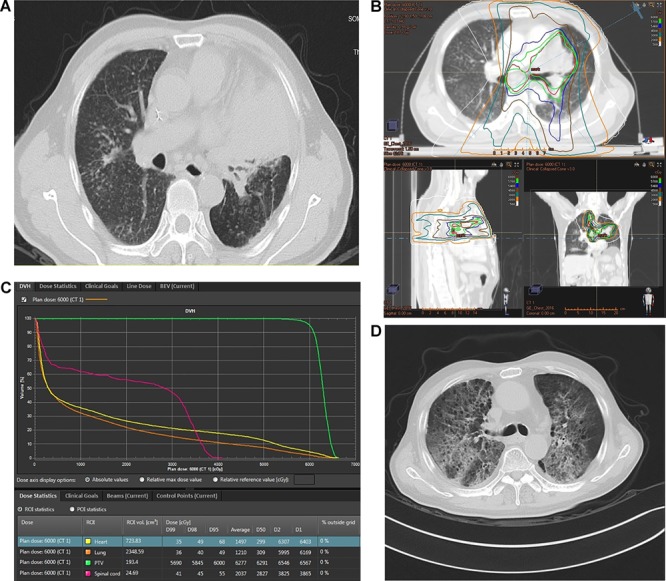
Silicotic nodules are distributed widely in both lungs, and a lung mass with corresponding atelectasis is seen in the left upper lung (**A**). The prescribed total dose was 60 Gy in 30 fractions once daily, five fractions per week (**B**). Dose–volume histogram (**C**). One patient developed grade 5 RP two months after chest radiotherapy (**D**).

The diagnosis of chronic silicosis is based on patients’ occupational exposure to silicon, symptoms and chest computed tomography (CT). The common occupations are miners, constructors, quarry workers, pottery workers and denim sandblasting workers. The symptoms of silicosis are atypical, including cough, fever, shortness of breath and chest pain. Chest radiography or CT scan was recommended for cases who had occupational exposure to silica particles to aid in diagnosis. The main manifestations were middle and lower pulmonary nodules (usually 1–3 mm in diameter), reticular shadows or large fusion lesions (shown in Fig. 1A).

### Radiotherapy

Palliative or radical radiotherapy were delivered through IMRT. Involved-field radiotherapy based on CT or Positron Emission Tomography/Computed Tomography (PET-CT) was delivered. The plan target volume (PTV) was defined as the clinical target volume (CTV) plus 5–10 mm to account for the daily set-up variation and respiratory movement. The prescribed total dose was 60 Gy in 30 fractions once daily, five fractions per week (shown in Figure 1B). Of all patients, 13 patients received cisplatin-based neoadjuvant or concurrent chemotherapy. Nine patients were treated with 2–4 cycles of neoadjuvant chemotherapy. Three to four weeks after chemotherapy, radiotherapy was initiated. Four patients were treated with concurrent chemoradiotherapy. Two patients received weekly paclitaxel and carboplatin chemotherapy, while two patients received tri-weekly pemetrexed and cisplatin-based chemoradiotherapy.

### Radiotherapy planning and dose–volume histogram analysis

The lung, heart and spinal cord were delineated as organs at risk. The following conditions were used to limit the dosage to the organs at risk: the MLD was of <20.0 Gy, V_20_ (volume receiving above 20 Gy) <30%, V_30_ (volume receiving >30 Gy) <20% and V_5_ (volume receiving >5 Gy) <60%, respectively. The V_40_ of the heart was <40%. The mean heart dose was <25.0 Gy. The maximum spinal cord dose was 45.0 Gy. The dose–volume histogram was obtained by Philips Radiation Oncology Systems (Pinnacle version 8.0, Figure 1C).

### Radiation-induced pneumonitis and overall survival

In this study, grade >2 acute RP was set as the primary endpoint. Acute RP was graded monthly according to the National Cancer Institute’s Common Terminology Criteria for Adverse Events (CTCAE), version 3.0. Body examination and chest CT scan were performed if the patient had symptoms as follows: cough, fever, dyspnea and chest tightness. Patients who had grade >2 RP received oxygen therapy and steroid treatment. The second endpoint of this study was OS. OS was calculated as the time from the date of the first day of treatment to death or censoring.

### Statistical analysis

All patients were divided into two groups according to the grade of RP: RP ≤2 and RP >2. The Independent t-test was used to analyze the difference of continuous variables between two groups. Survival curves were estimated by the univariate Kaplan–Meier method. All statistical calculations were performed with SPSS 13.0 for Windows (Chicago, IL). *P* < 0.05 was considered statistically significant.

## RESULTS

### Characteristics of patients

The characteristics of all patients who enrolled in this study are summarized in Table 1. Of all patients, 20 were male and two were female. The study cohort had a median age of 59 years (range: 48–75 years). The median time for all patients to be diagnosed with silicosis was 10 years, ranging from 3 to 30 years. The number of patients with lung squamous cell carcinoma, adenocarcinoma and small cell carcinoma was 14, 6 and 2, respectively. A total of 21 patients (95.5%) had a history of smoking and 14 (63.6%) of alcohol consumption. Four of 22 cases were stage II, 12 were stage III and six were stage IV. One month after radiotherapy, a CT-based clinical evaluation was performed for all patients. Two patients (9.1%) experienced complete response (CR), 11 (50.0%) partial response (PR) and 9 (40.9%) stable disease (SD).

**Table 1:** Patients’ characteristics

Characteristic	*n*	%
Age (years, range)	48–75	
History of silicosis (years, range)	3–30	
Pre-RT pulmonary function		
FEV1 (L), median	1.69	
FVC (L), median	2.28	
DLCO (mL min^−1^ mmHg^−1^), median	7.29	
CRP level		
<10 mg/L	17	77.3
≥10 mg/L	5	22.7
Sex		
Male	20	90.9
Female	2	9.1
Tumor location		
Left	13	59.1
Right	9	40.9
Histology		
Squamous cell	14	63.6
Adenocarcinoma	6	27.3
Small cell lung cancer	2	9.1
Smoking history		
Never	1	4.5
Ever	21	95.5
Alcohol history		
Never	8	36.4
Ever	14	63.6
PS score		
0	0	0
1	17	77.3
2	5	22.7
Clinical stage		
II	4	18.1
III	12	54.5
IV	6	27.3
Neoadjuvant chemotherapy		
No	13	59.1
Yes	9	40.9
Concurrent chemotherapy		
No	18	81.8
Yes	4	18.2
Response		
CR	2	9.1
PR	11	50.0
SD	9	40.9
RP classification		
≤2	15	68.2
3–5	7	31.8

### Pre-RT pulmonary function

Three patients had dyspnea before radiotherapy. The median FEV1 was 1.69 L. The range of FVC was from 1.26 L to 3.24 L (median: 2.28 L). The median DLCO was 7.29 mL min^−1^ mmHg^−1^. Most patients suffered from pulmonary ventilation and diffusive dysfunction. There were no significant differences in pulmonary function between patients with RP ≤2 and patients with RP >2 (*P*>0.05, Table 2). Serum C-reactive protein (CRP) level was borderline significantly higher in patients with RP >2 than in patients with RP ≤2 (10.14 mg/L vs 6.96 mg/L, *P* = 0.32, shown in Table 2).

**Table 2:** Comparison of pre-RT pulmonary function and dose–volume metrics according to RP

Variable	RP		*P*
	≤2	>2	
FEV1	1.57 ± 0.51	1.49 ± 0.50	0.72
FVC	2.27 ± 0.44	2.01 ± 0.50	0.34
DLCO	7.83 ± 4.44	7.33 ± 2.02	0.79
CRP level	6.96 ± 4.91	10.14 ± 9.89	0.32
V_5_ (%)	38.53 ± 10.40	53.51 ± 6.42	<0.01
V_10_ (%)	29.96 ± 8.84	39.46 ± 7.30	0.02
V_15_ (%)	24.05 ± 7.21	30.96 ± 7.30	0.04
V_20_ (%)	19.50 ± 6.45	24.60 ± 4.86	0.08
V_25_ (%)	14.86 ± 5.02	19.35 ± 6.78	0.15
V_30_ (%)	12.42 ± 5.21	15.74 ± 3.68	0.15
MLD (cGy)	1003 ± 300	1299 ± 200	0.03

### RP

Twenty patients completed the radiotherapy as planned. Two patients terminated all treatment 2 weeks after the initiation of radiotherapy due to intolerant aggravated dyspnea. Of the 22 patients, 10 patients (45.5%) developed RP ≥2: 3 (13.6%) grade 2, 4 (18.2%) grade 3, 1 (4.5%) grade 4, and 2 (9.1%) grade 5 (shown in Figure 1D). Two RP-related deaths (9.1%) occurred within 3 months after radiotherapy. In the univariate analysis, age, tumor location, alcohol history, performance score (PS) score, tumor stage, radiotherapy techniques, neoadjuvant or concurrent chemotherapy were not associated with RP (*P*>0.05). Dosimetric factors V_5_, V_10_, V_15_, V_20_ and MLD, were significantly higher in patients who had RP >2 (*P* < 0.05). In univariate analysis, V_25_ and V_30_ were not associated with RP (*P* > 0.05).

### RP and prognosis

**Fig. 2.**
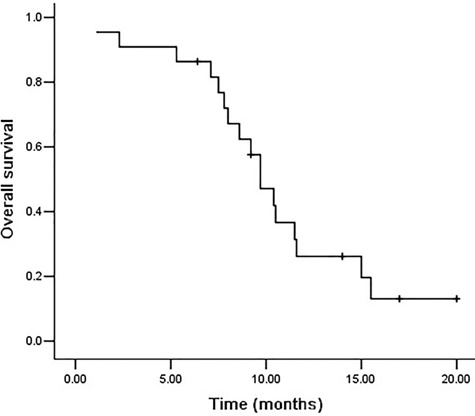
Overall survival curves in all patients, The 1- and 2-year overall survival rates weres 26.2 and 13.1%, respectively.

**Fig. 3.**
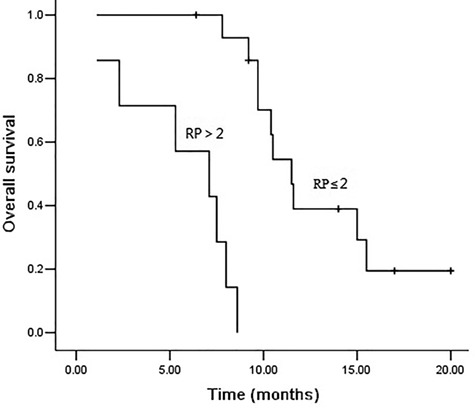
Overall survival curves according to RP; the median overall survival times in patients with RP ≤2 and RP >2 were 11.5 and 7.1 months, respectively.

The median follow-up time was 23.0 months. The 1- and 2-year overall survival rates were 26.2 and 13.1%, respectively (shown in Figure 2). Seventeen patients died during follow-up. Two patients died of lethal RP. Distant organ metastasis was observed in 10 patients. Patients’ age, tumor location, alcohol history, PS score, tumor stage, radiotherapy techniques, neoadjuvant or concurrent chemotherapy were not associated with OS (*P* > 0.05). RP ≤2 was associated with better OS (*P* < 0.05, shown in Figure 3).

## DISCUSSION

The relationship between chronic silicosis and lung cancer risk has been investigated for many years. In 2001, Steenland *et al*. [20] conducted a pooled exposure-response analysis focusing on the risk of lung cancer among 65 980 workers with silicosis, and a significant positive relationship was reported. The cumulative exposure to silica was a strong predictor of lung cancer (*P* = 0.0001). The exposure-response effect was reported, and an individual’s cumulative exposure threshold to silica was 1.8 mg/m^3^ [21]. Silica is an independent risk factor for lung cancer in addition to smoking. However, it is unclear whether silicosis has a negative effect on the anti-cancer treatment of lung cancer when patients with silicosis develop lung cancer. It is well known that most patients with silicosis have pulmonary diffusive dysfunction before hospitalization [19]. To examine the impact of the safety and efficacy of radiotherapy in lung cancer patients with concomitant silicosis, we conducted this retrospective study. To the best our knowledge, this is the first study to investigate pulmonary toxicity after radiotherapy in patients with chronic silicosis.

In this study, severe toxicity was observed even when we delivered a lower dose of radiotherapy than that delivered in a previous study [22], and most patients did not have chemotherapy. Two patients terminated all treatment 2 weeks after the initiation of radiotherapy due to acute exacerbation of silicosis. Seven patients (31.8%) developed RP ≥3. Two RP-related deaths (9.1%) occurred within 3 months after radiotherapy. Dosimetric factor V_5_ was significantly higher in patients who had RP>2 (*P* < 0.05). This result was consistent with the previous report. Allen *et al.* [23] treated mesothelioma patients with IMRT after extrapleural pneumonectomy (EPP) and adjuvant chemotherapy. That study showed that nearly half of the patients developed fatal acute pneumonitis after radiotherapy. High incidence of pneumonitis may be due to a high volume of lung irradiated with the low dose. Therefore, IMRT should be used cautiously for lung cancer patient with a large volume of radiation field.

The clinical response evaluation in this study showed that the objective response rate (ORR) was 59.1%, comparable to other reported trials using radiotherapy alone [24, 25]. In our study, The 1- and 2-year overall survival rates were only 26.2 and 13.1%, respectively. RP ≤2 was associated with better overall survival (*P* < 0.05). The majority of lung cancer patients with chronic silicosis died after radiotherapy, not because of uncontrolled tumors but because of severe lung injury induced by X-ray.

Minegishi *et al*. conducted a phase I study to investigate the safety and efficacy of chemotherapy in metastatic small cell lung cancer patients with idiopathic pulmonary fibrosis [26]. Although one patient had an acute exacerbation of fibrosis due to chemotherapy, the median overall survival time was 8.7 months, equivalent to that observed in patients without lung fibrosis. In our study, nine patients received neoadjuvant chemotherapy, and no significant increase in toxicities was observed during chemotherapy. Therefore, chemotherapy was safe compared with X-ray radiotherapy. Moreover, for patients with chronic silicosis, we need to choose chemotherapeutic drugs with less pulmonary toxicity as far as possible and avoid using drugs with strong pulmonary toxicity such as gemcitabine [27] and docetaxel [28].

Dose–volume parameters, such as V_5_, V_20_, V_30_ and MLD, are common predictors of RP. However, the most accurate dosimetric parameter has not been established for lung cancer patients with poor pulmonary function. In this study, all patients’ radiotherapy plans met The National Comprehensive Cancer Network (NCCN)-recommended dose–volume constraints, but the incidence of RP was 45.5%. This is much higher than that in patients without silicosis [29]. This suggests that the current NCCN recommendation might not be suitable for patients with poor pulmonary function [14, 30]. Further prospective clinical studies are required to confirm these findings.

## CONCLUSIONS

In summary, this retrospective study showed that radiotherapy is associated with a high incidence of lethal RP in lung cancer patients with chronic silicosis. RP is related to OS.
